# Salvage surgery for mesenteric lymph node metastasis by resection of the first jejunal flap and reconstruction with the second jejunal flap

**DOI:** 10.1093/jscr/rjad686

**Published:** 2023-12-28

**Authors:** Daiki Kitano, Kazunobu Hashikawa, Tatsuya Furukawa, Tadashi Nomura, Kotaro Tamagawa, Shunsuke Sakakibara, Ken-ichi Nibu, Hiroto Terashi

**Affiliations:** Department of Plastic and Aesthetic Surgery, Kobe University Graduate School of Medicine, Kobe, Japan; Department of Plastic and Reconstructive Surgery, Nagoya University Graduate School of Medicine, Nagoya, Japan; Department of Otolaryngology-Head and Neck Surgery, Kobe University Graduate School of Medicine, Kobe, Japan; Department of Plastic and Aesthetic Surgery, Kobe University Graduate School of Medicine, Kobe, Japan; Department of Otolaryngology-Head and Neck Surgery, Kobe University Graduate School of Medicine, Kobe, Japan; Department of Plastic and Aesthetic Surgery, Kobe University Graduate School of Medicine, Kobe, Japan; Department of Otolaryngology-Head and Neck Surgery, Kobe University Graduate School of Medicine, Kobe, Japan; Department of Plastic and Aesthetic Surgery, Kobe University Graduate School of Medicine, Kobe, Japan

**Keywords:** mesenteric lymph node metastasis, jejunal flap, oropharyngeal cancer, lymphatic flow alteration, mesenteric lymphadenectomy, total pharyngolaryngectomy

## Abstract

We report a case of a second free jejunal transfer to treat metastasis in the mesenteric lymph node of the first jejunal flap. A 73-year-old man underwent total pharyngolaryngectomy, bilateral neck dissection, and free jejunal transfer for recurrent hypopharyngeal cancer [left pyriform sinus, pT2N0, moderately differentiated squamous cell carcinoma (SCC)] after radiotherapy. Seven years post-surgery, he underwent transoral videolaryngoscopic surgery for oropharyngeal cancer (soft palate, pT1N0, well-differentiated SCC). Ten years after the first jejunal transfer, metastasis was found in the mesenteric lymph node surrounding the jejunal flap’s vascular pedicle. Under general anesthesia, resection of the first jejunum including the affected lymph node, and second jejunal transfer were performed. Lymph node pathological examination revealed poorly differentiated SCC, compatible with pharyngeal cancer metastasis. After neck dissection and jejunal flap transfer, lymphatic collateral pathways toward the flap’s mesenteric lymph node might form. Possibly, hypopharyngeal or oropharyngeal cancer metastasized via this pathway.

## Introduction

Free jejunal transfer is a common reconstruction method after total pharyngolaryngectomy (TPL) [1]. It results in early oral intake resumption and reduces the incidence of fistula formation and lumen stenosis [2]. Although jejunal flap harvesting requires laparotomy, it is associated with an acceptable complication rate. The incidence of postoperative bowel obstruction is 2–4% [2, 3]. Moreover, if the first jejunum fails, another jejunal flap can be harvested and transplanted again (i.e. second jejunal transfer) [4].

The jejunal flap receives blood flow from the jejunal artery, a branch of the superior mesenteric artery coursing along the mesenteric margin and forming a vascular network that nourishes the serosa, muscle layer, and mucosa. The jejunal artery and concomitant veins are recruited for vascular anastomosis during free jejunal transfer. Lymphatic vessels travel along the jejunal artery via the mesenteric lymph nodes, and flow into the para-aortic lymph nodes [5]. We report a rare case of metastasis to the mesenteric lymph node of the first jejunal flap with a literature review.

## Case report

A 73-year-old man underwent TPL, bilateral neck dissection (level II–IV and bilateral lateral retropharyngeal lymph nodes), and free jejunal transfer for recurrent hypopharyngeal cancer [left pyriform sinus, pT2N0, moderately differentiated squamous cell carcinoma (SCC)] post-radiotherapy. The pedicle of the jejunal flap was anastomosed to the right transverse cervical artery and external jugular vein. Seven years postoperatively, he underwent transoral videolaryngoscopic surgery (TOVS) for oropharyngeal cancer (soft palate, pT1N0, well-differentiated SCC).

**Figure 1:**
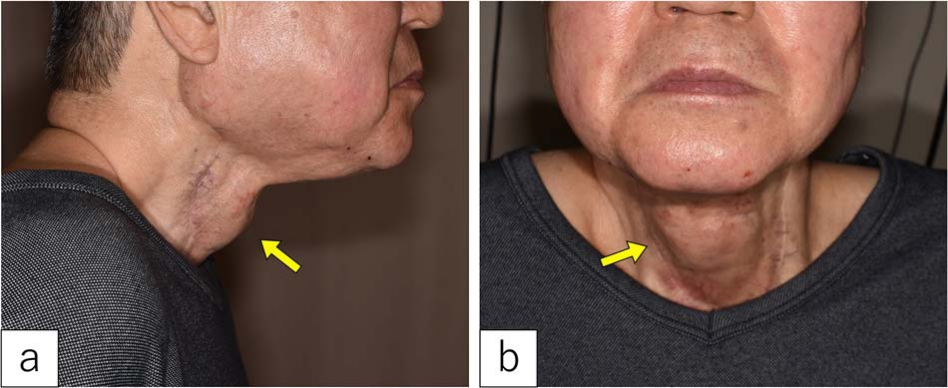
On admission. A 3-cm-sized cervical subcutaneous tumor was confirmed (arrow).

**Figure 2:**
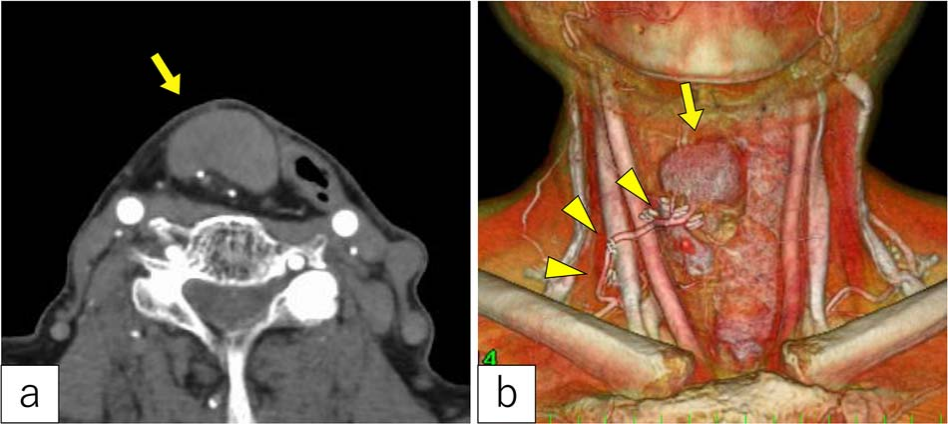
Radiological findings. (a) Computed tomography revealed a tumor under the cervical flap (arrow). (b) The tumor involved the vascular pedicle of the jejunal flap (arrowhead).

Three years post-TOVS, a mass was noted in the anterior neck (Fig. 1). Computed tomography (CT) revealed lymphadenopathy in the mesenteric lymph node of the transferred jejunal flap, involving the flap’s vascular pedicle (Fig. 2). Ultrasonography-guided fine-needle aspiration cytology revealed atypical nuclei compatible with SCC metastasis.

**Figure 3:**
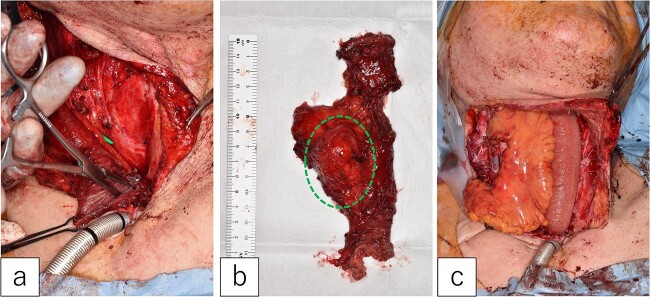
Second jejunal transfer. (a) There were no lesions in the lumen of the first jejunum. (b) The tumor was confined to the mesentery (dotted circle). (c) The first jejunum was replaced with the second jejunum.

The first jejunal flap was removed under general anesthesia. A mild scar formed between the jejunal flap and cervical tissues, but no pathological adhesions occurred. The mesenteric tumor was confined to the lymph node and did not extend into the jejunal lumen (Fig. 3a and b).

A new jejunal flap was harvested and used to replace the first jejunum (Fig. 3c). The recipient vessels were the right transverse cervical artery and internal jugular vein. The flap was monitored every 4 h for 5 days post-surgery. The postoperative course was uneventful, except for minor pharyngo-cutaneous fistula formation, which improved with local treatment.

**Figure 4:**
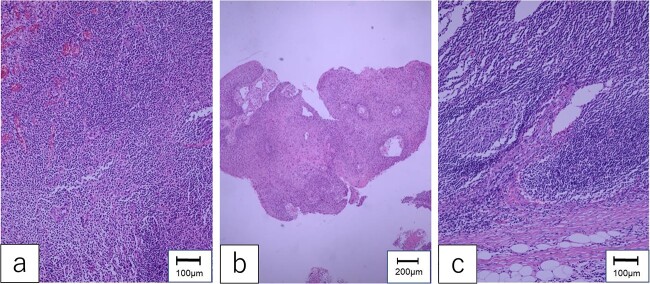
Pathological findings. (a) The specimen of hypopharyngeal cancer showed that dysmorphic cells with well-defined nucleoli were proliferating without polarity, diagnosed as moderately differentiated SCC. (b) The specimen of oropharyngeal cancer was compatible with well-differentiated SCC, which formed cancer pearls and papillary structures. (c) The mesenteric tumor of the first jejunum confirmed poorly differentiated SCC, which consisted of dysplastic cells with rounded nuclei and distinct nucleoli.

Pathological examination of the mesenteric tumor revealed a poorly differentiated SCC in the lymph node without extra-nodular extension (Fig. 4). There was no evident lymphatic vessel infiltration or skip lesions near the pedicle of the first jejunal flap. Determining the origin of the metastasis was difficult. The patient was discharged 6 weeks postoperatively. Two years after the salvage surgery, he died of multiple distant metastases.

## Discussion

To date, there are three case reports on metastasis to the mesenteric lymph node of the jejunal flap [6–8]. These cases were diagnosed within 3 years after the first surgery; the authors concluded that the residual primary cancer metastasized to the mesenteric lymph node of the first jejunum via lymphatic flow. Here, 10 years had passed since the first surgery. Although the lymph node metastasis in the mesentery was SCC consistent with hypopharyngeal cancer, a metastatic lesion took long to form after the first surgery. Additionally, no residual or recurrent cancer was found on the first jejunal flap’s pharyngeal or esophageal side. Therefore, the oropharynx was considered the origin of the metastasis.

Lymphatic flow is altered by collateral pathway formation after lymph node dissection [9]. Here, in addition to the bilateral neck dissection, a new lymphatic collateral pathway toward the mesenteric lymph nodes of the jejunal flap might have formed along the vascular pedicle of the jejunal flap. We hypothesized that oropharyngeal cancer metastasized to the mesenteric lymph nodes of the first jejunum via this altered lymphatic flow.

Another possible explanation of the SCC in the mesenteric lymph node is a primary cancer developing in the first jejunal flap’s mesenteric lymph node. However, primary mesenteric cancer is rare, with a reported incidence of 0.012% [10]. Additionally, > 90% of primary mesenteric tumors are sarcomas. Only one study reported SCC detected as a primary mesenteric tumor [11].

The third possibility is a metastasis of a primary microscopic cancer developing in the remaining pharyngeal mucosa. Although no suspicious could be detected by endoscopy, CT, or positron emission tomography (PET) within 2 years after the salvage surgery, heterochronous multiple pharyngeal SCCs and primary unknown lymph node metastasis are common in Japanese drinkers with heterozygous dehydrogenase [12].

Mesenteric lymphadenopathy in the transferred jejunum is common. Makimoto et al. reported that 10/15 patients had enlarged mesenteric lymph nodes (>3 mm) after free jejunal transfer [13]. Lymphadenectomy was performed in three patients with >10 mm lymph nodes, but none had malignant tumor metastases. Their findings were compatible with reactive lymphadenopathy, and the transferred jejunum responded to a new environment different from the abdominal cavity. Suzuki *et al.* found that 43/72 patients who underwent free jejunal transfer had mesenteric lymphadenopathy (>5 mm) [14]. Of 13 patients with increased size, 1 had metastasis to the mesenteric lymph nodes. Careful monitoring using ultrasonography, enhanced CT, and/or PET should be considered for enlarging mesenteric lymphadenopathy of the transferred jejunum.

No established treatment strategy exists for metastasis in the mesenteric lymph node of the first jejunal flap. Previously, lymph node metastasis was treated by lymphadenectomy [6–8]. However, one patient died 4 months after the salvage because of multiple metastases [8]. Here, we resected the first jejunal flap with the mesentery and performed reconstruction with the second jejunal flap. No additional treatment was conducted since bilateral neck dissection and adjuvant radiotherapy had already been performed. Eventually, the patient died of multiple distant metastases; he had survived for 2 years after the salvage surgery. The treatment plan should be determined individually in patients. In conclusion, lymphatic collateral pathways may form toward the jejunal flap’s mesenteric lymph nodes after bilateral neck dissection and jejunal transfer, which can lead to metastasis. Although mesenteric metastases are rare, careful monitoring is required after free jejunal transfer.
